# Complement Component C3: A Novel Biomarker Participating in the Pathogenesis of Non-alcoholic Fatty Liver Disease

**DOI:** 10.3389/fmed.2021.653293

**Published:** 2021-07-29

**Authors:** Juqiang Han, Xiang Zhang

**Affiliations:** ^1^Institute of Liver Disease, The 7th Medical Centre of Chinese People Liberation Army General Hospital, Beijing, China; ^2^The Department of Medicine and Therapeutics, State Key Laboratory of Digestive Disease, Institute of Digestive Disease, Li Ka Shing Institute of Health Sciences, Chinese University of Hong Kong Shenzhen Research Institute, The Chinese University of Hong Kong, Hong Kong, China

**Keywords:** non-alcoholic fatty liver disease, complement C3, complement C3 receptor, pathogenic mechanism, therapy

## Abstract

Non-alcoholic fatty liver disease (NAFLD) is currently the most common cause of chronic liver disorder worldwide. The pathological spectrum of NAFLD ranges from simple steatosis to non-alcoholic steatohepatitis (NASH) that induces progressive liver cirrhosis and eventually hepatocellular carcinoma (HCC). However, the molecular mechanisms driving the transformation of NASH are obscure. There is a compelling need for understanding the pathogenic mechanisms of NASH, and thereby providing new insight into mechanism-based therapy. Currently, several studies reported that complement system, an innate immune system, played an important role in the pathogenesis of NAFLD, which was also proved by our recent study. Complement component 3 (C3), a protein of the innate immune system, plays a hub role in the complement system. Herein, we present a review on the role and molecular mechanism of C3 in NASH as well as its implication in NASH diagnosis and treatment.

## Introduction

Non-alcoholic fatty liver disease (NAFLD) has become a very common liver disease worldwide. The disease spectrum includes non-alcoholic fatty liver (NAFL), non-alcoholic steatohepatitis(NASH), nutritional fibrosis and hepatocellular carcinoma Cancer (HCC). Epidemiological investigations show that the prevalence of NAFLD is 25–30% in western countries (1). With the change of diet structure and lifestyle, the prevalence of NAFLD is 17–46% in China (2). Notably, the prevalence of NAFLD in young children is currently increasing year by year, and the high prevalence rate is alarming (3). Among NAFLD patients, the prevalence of NASH is about 20% (4). However, the diagnosis of NASH relies on liver biopsy and the non-invasive diagnostic methods are limited. To date, there's no Food and Drug Administration (FDA)-approved drug for NAFLD and NASH treatment. Therefore, there is an unmet clinical need for the diagnosis, prevention and treatment of NAFLD and NASH. In recent years, innate immunity is thought to play an important role in the development of NAFLD. Therefore, we summarized previous studies and provided a holistic framework concerning the relationship between complement and NAFLD.

## The Overview of Complements System

**Figure 1:**
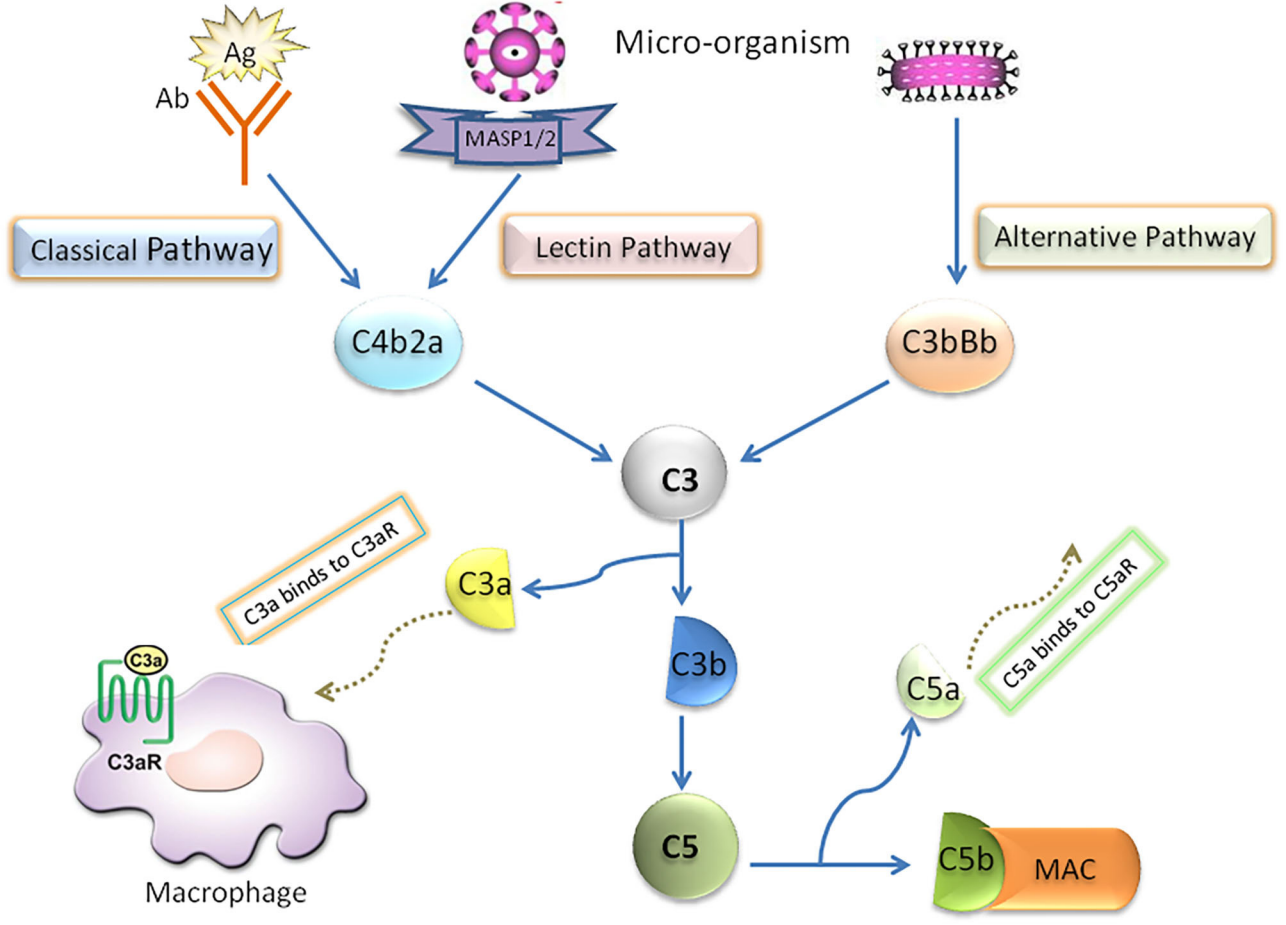
Schematic diagram of complement system activation.

In the early stage of NAFLD, bacterial endotoxin, free fatty acid (FFA), cholesterol and many other substances in the body can activate the complement system through danger associated molecular patterns (DAMPs) or pathogen associated molecular pattern (PAMPs) (5, 6). Complement system, which is considered as an important innate immunity (7–10), has been confirmed to be cascade-activated through the following three pathways: classical, lectin and alternative, all of which converge in the formation of fraction C3. Briefly, in the activated complement signaling pathway, complement component C3 is cleaved into C3a and C3b through C3 converting enzyme, in which C3b binds with C3 converting enzyme complex to form C4bC2aC3b complex in classical pathway and lectin pathway and C3bBbC3b complex in alternative pathway. Both complexes are converting enzymes of complement molecule C5, and then further activate the downstream molecules of Complement system to form membrane attack complex (MAC/C5b-9). Furthermore, the classical activation pathway mainly involves the binding of antigen with immunoglobulin (IgM or IgG) or C-reactive protein. In the lectin pathway, Complement activation is triggered by the interaction complex carbohydrate residues with the surface of pathogens to circulating mannose binding lectins (MBL) or ficolins. The alternative pathway is activated by the direct combination of hydrolyzed C3b and bacterial membrane surface (Figure 1).

## Complement C3 and NAFLD

Currently, the role of Complement system in NAFLD disorders has been extensively investigated in clinical epidemiological studies (11). Serum complement C3 levels are positively associated with the severity of NAFLD. A Turkish case-control study involving 46 NAFLD patients demonstrated that the level of Complement component C3 was significantly higher in peripheral blood of NAFLD patients than that of healthy control group and chronic hepatitis B control group (12). Moreover, a Dutch cross-sectional study involving 523 middle-aged and elderly patients with NAFLD found that the level of C3a, the active product of complement C3, was closely related to liver fat content (13). Consistently, two large sample epidemiological studies recruited thousands of cases in China showed that the level of serum complement C3 was an independent risk factor for the diagnosis of NAFLD and related to the prevalence and the severity of NAFLD (14, 15). Apart from the serum, the deposition of C3 is also identified in the liver tissue of NAFLD patients accompanied with the deposition of MAC-C9 (16). The deposition of C3 was proved to be mainly located around the hepatocytes with macrovesicular steatosis. Subsequently, clinicopathological examination confirmed that a large number of hepatic parenchymal cells were apoptotic in the liver tissue with complement C3 activation. Complement C3 activation could lead to a large number of neutrophils infiltration and abnormal increase of IL-8 and IL-6 expression in liver tissue, while C9 deposition could lead to increased IL-1β expression in liver cells. Additionally, in other patients with NAFLD, there was a close correlation between serum C3 level and NAFLD. For example, Pan et al. demonstrated that C3 was the only highly predictive factor in diagnosing NAFLD from 648 recruited patients with chronic kidney disease by Logistic regression analysis (17). Ursini et al. recruited 164 patients with rheumatoid arthritis, of which 25% (41/164) were complicated with NAFLD. Further logistic regression analysis also confirmed the high correlation between C3 and NAFLD analysis (18). Most importantly, Himoto et al. found that the increased serum C3 levels are closely related to the abnormal metabolism of the body including obesity, insulin resistance, and/or hepatic steatosis in those patients with chronic hepatitis C, which had nothing to do with chronic HCV infection (19). Collectively, the complement system is closely related to NAFLD. It is speculated that the complement system is largely activated to regulate the immune inflammatory response in the pathogenesis of NAFLD, which directly participates in the occurrence, development and prognosis of NAFLD.

## The Pathogenic C3 Activation in NAFLD Progression

**Figure 2:**
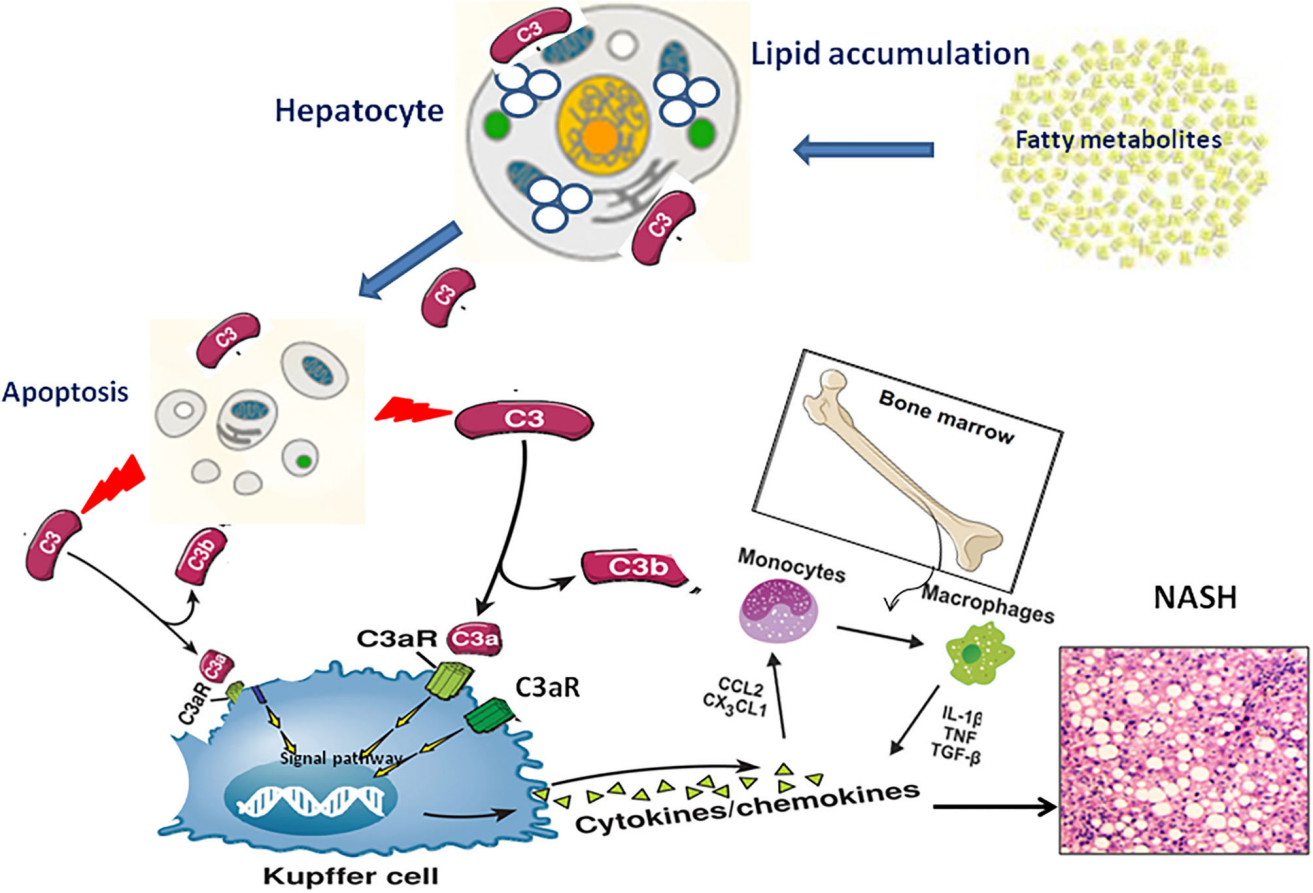
The pathogenic mechanism of C3 activation in NAFLD progression through lipid accumulation, immune response and apoptosis.

NAFLD severity was closely associated with accumulation of activation products of C3 around steatotic hepatocytes. However, the underlying mechanism by which complement C3 in NAFLD remained elusive. Hepatocytes are confirmed to be the predominant origin of complement components including C3 protein. Because C3 is the key molecule in the pathway of complement system activation (7), several hypotheses are proposed that C3 plays an important role in lipid metabolism in the pathogenesis of NAFLD (Figure 2): Firstly, C3 is mainly synthesized by hepatocytes and identified to appear in lipoprotein particles such as high density lipoprotein and chylomicron (20, 21). When complement C3 gene was knocked out in mice, serum triglyceride levels increased 58% higher compared with wild-type mice, and the increased lipoprotein profile is mainly low-density lipoprotein and very low-density lipoprotein (21), indicating a potential role of C3 in lipid metabolism regulation (22). Secondly, Complement system directly regulates the oxidative stress in hepatocytes with excessive fat accumulation. It is well-known that multiple-hit hypothesis has been widely accepted in the pathogenesis of NAFLD. The first hit is closely linked with insulin resistance under fat accumulation. With the response of hepatocytes to oxidative stress, a large number of inflammatory cytokines are secreted in the liver, which further cause the second hit in hepatocytes (23). Complement component C3, as a major player in innate immune response, might be activated by the first hit and forming the second hit in NAFLD pathogenesis (24). Thirdly, the phenomenon of apoptosis is a typical pathological feature in the liver with NAFL and NASH. Due to the accumulated fat droplets in hepatocytes, a variety of apoptotic cascade pathways are activated including caspases 3 and 7 or cleavage of cytokeratin 18, resulting in a large number of hepatocytes apoptosis. Complement system was found to be quickly activated by the apoptotic hepatocyte debris. Furthermore, the activated Complement system immediately recognized and cleaned the apoptotic pathological liver cells, thus maintaining the homostasis in liver (25). Thus, elevation of serum complement C3 might act as a protective response in NAFLD mediated by apoptosis. Finally, Complement activation is indicated to be involved of novel molecular mechanism in the pathogenesis of NAFLD. Acylation-stimulating protein (ASP), a C3 derivative involved in adipocyte lipid metabolism by stimulating triglyceride synthesis, was reported to be increased in NAFLD patients (26). A vicious cycle has been further confirmed in the pathogenesis of NAFLD. ASP can promote the fat accumulation in liver cells to exacerbates hepatic steatosis. On the other hand, the fatty liver promotes the activation of complement system to increase ASP synthesis (12). In brief, there is a potential balance in the pathogenesis of NAFLD between complement system activation and hepatocyte lipid metabolism signaling, which maintains the stability of liver internal environment. Once the balance is broken, the process of hepatocyte lipid metabolism will enter a vicious circle, which exacerbates irreversibly hepatic steatosis as a consequence.

## C3a/C3aR Activation and Metabolic Function

Recently, increased evidence showed that C3a can change the storage, transportation and utilization of glycolipids in adipocytes and directly affect adipogenesis, glucose uptake and lipolysis in the pathogenesis of NAFLD. Oral administration of selective C3aR antagonist for 8 weeks can significantly improve the typical symptoms of metabolic syndrome in diet-induced obese rats, including weight loss, visceral fat reduction, glucose and insulin intolerance improvement, adipose tissue inflammation relief, blood lipid concentration dropping, etc. The above research results innovatively provide two novel mechanisms of C3a involved in energy metabolism on theoretical and experimental basis, that is, C3a can not only promote the uptake of fatty acids and glucose by adipocytes, but also inhibit fat burn-off by inhibiting cAMP synthesis and lipolysis in adipocytes (27). These studies fully elucidate the important correlation between complement C3a/C3aR signaling pathway and energetic metabolism, and further reveal the molecular mechanism of abnormal immune response aggravating obesity and metabolic dysfunction. More importantly, antagonists targeting C3a/C3aR signaling pathway is considered as a novel strategy for the treatment of metabolic dysfunction, including NAFLD. Consistently, our recent result also found that C3a/C3aR signal pathway was closely related to the development of NASH-fibrosis. In C3aR^−/−^ mice model, we also demonstrated that C3aR depletion significantly reduced the progression of NASH related liver fibrosis (28).Studies have confirmed that C3a is an important derivative produced by C3 cleavage when complement system is activated. C3a has significant biological characteristics of anaphylactic toxin and is an important pro-inflammatory molecule in the body. It can directly trigger mast cells degranulation, inflammatory reaction, chemotaxis effect, granulocyte activation as well increasing vascular permeability, promoting smooth muscle contraction and clearing away the immune complexes, etc (29). In other words, C3a plays an important role in the pathogenesis of a variety of clinical diseases, including organ ischemia-reperfusion injury, sepsis and metabolic inflammation (27, 30–37). Structurally, C3a is composed of 77-aa polypeptide containing three to four helical regions. A series of irregular amino acid residues is also proved in the C-terminal of C3a protein, which is flexible in spatial conformation. There is evidence that these flexible C-terminal residues are necessary to stabilize the conformation on binding C3aR by the upstream a helix (38–40). It has been demonstrated that C3a exerts its biological effect mainly by binding to its receptor C3aR, which belongs to G protein-coupled receptor containing seven trans-membrane regions. C3a/C3aR interaction is generally regulated by G protein-coupled receptor kinase-mediated receptor phosphorylation (41). In the past, it has been considered that C3aR is the only specific receptor of C3a. It shares close homology with C5a specific receptors C5aR1 and C5aR2. However, recent studies have showed that the interaction of C3a/C3aR seems to be more complex than expected. For example, C3aR has been found to be able to couple with heterotrimeric G proteins depending on different cell types (42–44). In human granulocytes, C5a can inhibit the activity of C3aR. Ruan et al. confirmed that C3a can form a complex with CpG oligonucleotides to improve the release of IFN-a in monocytes (45). Neuropeptide TLQP-21 (a cleaved fragment of VGL propeptide) was found to specifically binds on C3aR and fully activate the biological function of C3aR in mice, which completely comply with the conformational change of ligand/receptor interaction (46, 47). Additionally, recent studies have demonstrated that C3a can bind to the receptor of advanced glycation end products (RAGE) with very high affinity, but this high affinity interaction cannot be explained by a simple ligand upon receptor binding (45, 48).

## C3a/C3aR Axis and Immune Response in NAFLD

### Kupffer Cells/Macrophages and C3a/C3aR Axis in NAFLD

Liver is the largest reservoir of macrophages in the body, and macrophages in liver play a key role in the pathogenesis of NAFLD (23, 49, 50). According to different origin, macrophages in liver can be divided into two types, one is Kupffer cells fixed in liver, the other is monocytes/macrophages derived from bone marrow. They mainly play the role of innate immunity such as phagocytosis and secretion of inflammatory cytokines. At the early stage of NAFLD, Kupffer/macrophage cells are the first defense lines against the accumulation of excessive lipid metabolites in the liver. Firstly, steatotic hepatocytes disturb hepatic sinusoidal perfusion because of the “gap occupying” effect and Kupffer cells are subsequently attracted by neutrophils to the sinusoidal gap to participate inflammation. Secondly, free fatty acids (FFA) excessively interact with the FFA specific receptors on Kupffer/macrophage cells surface to regulate inflammatory response. Thirdly, Kupffer/macrophage cells mistakenly attribute the abnormal hepatocytes full of excessive lipid accumulation to the harmful substances and immediately phagocytize and destroy them, which further aggravate hepatocyte damage (51). Under the above conditions, these overactivated Kupffer/macrophage cells secrete a large number of inflammatory cytokines (such as TNFα, IL-6, IL-1β) as well as inflammatory chemokines (such as CCL2, CCL3, CCL5, CXCL16, CX3CL1,). In addition, Kupffer cells also recruit a large number of bone marrow-derived monocytes/macrophages into the liver to expand the inflammatory response and accelerate the liver from simple steatosis to NASH (52). CCL2/CCR2 interaction is proved to be the first signal pathway in Kupffer/macrophage recruiting monocytes from bone marrow (53). Afterwards, some other chemokines signal pathways are also confirmed through Kuppfer cells mechanisms such as CXCL10/CXCR3, CCL5/CCR1, and CCL1/CCR8 (11). In recent years, a number of research groups have got the highly consistent results by various ways to delete macrophages in NAFLD mice model (54–56). Namely, knocking-out monocytes/macrophages can reduce significantly the severity of liver steatosis and inflammation and further delay the process of NAFLD. However, so far, there is no systematic and in-depth study on the specific molecular mechanism of regulating macrophage activation in the whole pathogenesis of NAFLD.

As mentioned above, hepatocytes are the main origin to synthesize C3a molecules, and C3aR is predominantly located in the cell membrane of monocytes/macrophages. In the chronic phase of liver inflammation, C3a/C3aR signaling activity is showed more significant in monocytes/macrophages than that of neutrophils (57, 58). Under this condition, C3a can activate the signal pathway of peripheral blood monocytes with the co-stimulation of TLR-4, and further induce the secretion of various proinflammatory factors such as IL-1 β, TNF-α, IL-6 and PGE2 (59–63). This view is supported by evidence that liver steatosis have modest pathology reductions in C3aR1^−/−^ mice models (57). Therefore, we speculate that the C3a/C3aR interaction may be a novel signaling pathway by activating macrophages to regulate the occurrence and development of NAFLD. In the early stage of NAFLD, a large amount of fat accumulates in hepatocytes, resulting in the broken balance between complement system activation and hepatocyte lipid metabolism. Subsequently, Complement cascades enter into an overactivated state and excessive amount of C3 molecular is cleaved to release C3a. Through the specific interaction of C3a/C3aR, Kupffer/macrophages cells in the liver are directly activated to secrete inflammatory chemokines to recruit monocytes from peripheral blood into the liver, which further expand the inflammatory response in the steatotic liver (24) (Figure 2).

### Hepatic Stellate Cell and C3a/C3aR Axis in NAFLD

Under normal physiological conditions, component C3 is mainly expressed in hepatic parenchymal cells. However, recent evidence has demonstrated that C3 is also slightly expressed in other type cells such as bone marrow cells, lymphocytes, fibroblasts and endothelial like cells (64). Activated hepatic stellate cells, as the initiating factor of hepatic fibrogenesis, have obvious characteristics of fibroblasts. Up to now, there are relatively few reports about complement C3 directly involved in the activation of hepatic stellate cells (HSCs) about NAFLD. A recent study showed that activated hepatic stellate cells could promote the hepatocarcinogenesis through C3 signaling pathway by inhibiting the proliferation of CD4 + and CD8 + cells, promoting the exhausting anti-tumor T lymphocytes as well as improving the differentiation of bone marrow-derived suppressor cells (MDSC) (65). Consistently, our recent results demonstrated that C3a/C3aR signaling pathway was activated in the mouse model of nutritional fibrosis. The related mechanism is being further explored in C3aR^−/−^ mice (28). However, Component cascade activated C5 molecular is found to be a key factor that contributes to hepatic fibrosisgenesis by enhancing the migration ability of hepatic stellate cells. Furthermore, the C5a/C5aR axis was shown to directly mediate inflammatory, chemotactic and anaphylatoxic properties in innate and adaptive immunity as well as to modulate activation and migration of HSCs (66).

### T Cell and C3a/C3aR Axis in NAFLD

It has been confirmed that T cells play an important regulatory role in the pathogenesis of NAFLD. The balance has a directly effect in the pathogenesis of NAFLD between Th1-secreted pro-inflammatory cytokines and Th2-secreted anti-inflammatory cytokines (67). The number of CD4^+^ helper T cell 17 (Th17) subsets was significantly higher than that of regulatory T cells (Treg) in the pathogenesis of NAFLD (68–72). With the development from simple steatosis to NASH, the number of Th17 cells in liver and peripheral blood increased continuously in a clinical epidemiological study of 104 human subjects (including 30 patients with NASH, 31 patients with simple steatosis and 43 healthy controls), resulting in a significant increase in Th17/resting T regulatory cell ratio (70). There is no doubt that C3a molecule directly participates in T cells proliferation and differentiation as well as regulates the biological function, but the mechanism is complex (73, 74). It is still controversial whether C3aR is widely expressed in T cells (75). When C3aR gene was knocked-out as a target, the number of T cells was significantly reduced in C3aR^−/−^ mice model (73, 76). Further studies confirmed that increasing the intracellular expression of C3a in T cells can significantly prolong their survival (77, 78). TCR stimulation can significantly up-regulate the expression of C3aR mRNA in T cells (79). Therefore, it is speculated that C3a further regulates T cells proliferation, differentiation and biological functions through a potential autocrine way. Recent studies have shown that C3a can promote T cell proliferation, enhance T cell reaction and prolong inflammatory response by inhibiting Treg production (73, 76). After adoptive transfer of C3aR^−/−^ T cells into wild-type animals, the immunological function of Treg was seriously changed (76). In addition, there is evidence that C3aR activation in antigen-presenting cells (APCs) can inhibit Th2 polarization and further block IL-4 secretion (80). Under the synergistic effect of C5aR1 signaling pathway, C3aR can suppress the production of TGF -β1 by dendritic cells, reduce the stimulation of Treg differentiation, and then eliminate the inhibitory response to Th1 (76).

### Neutrophil and C3a/C3aR Axis in NAFLD

Neutrophil infiltration is usually observed in the liver of NAFLD patients, and the severity of infiltration is closely related to disease progression (81, 82). The excessive fat accumulation overloads the normal metabolic capacity of hepatocytes. Subsequently, the abnormal metabolic injury results in neutrophils to be overactivated and recruited to the steatosic liver. After administration the neutrophil-specific antibody 1A8 into mice, hepatic lipid accumulation and inflammation were significantly attenuated in HFD diet induced NAFLD models, which further slowed down the progress of NASH (83). Currently, neutrophil to lymphocyte ratio (NLR) has been clinically used as an effective biomarker to predict the severity of NAFLD (84–86). Accumulating evidence shows that NLR is positively correlated with NAFLD activity score (NAS) and an independent predictor of NAFLD prognosis. The higher the NLR value, the higher the severity of the disease, and the worse the prognosis of NAFLD. In the study of 101 NASH patients, the NLR value was significantly higher than that of controls without NAFLD (mean 2.5 vs. 1.6, *P* < 0.001) (87). In the stage of NASH related fibrosis, the NLR value in advanced fibrosis stage (f3-4) was significantly higher than that of patients with early fibrosis stage (f1-2) (median: 2.9 and 1.8, *P* < 0.001) (87). So far, it has been controversial for C3a/C3aR interaction in neutrophils because the pure neutrophils was isolated difficultly over a long period in the past, which resulted in the doubt that C3a induced neutrophil activation may be contaminated by non-neutrophils (88). At present, C3aR is definitely identified to express with high level on the surface of neutrophils. Curiously, although C3a/C3aR interaction can activate downstream ERK1/2/Akt signaling pathway, C3a alone does not play chemotactic function as well as stimulate neutrophil degranulation. Recent study suggested that the signaling produced by C3a stimulation of neutrophils was found to be dependent under the synergistic effect of C5aR2 (89). Another study demonstrated that C3a directly prevents neutrophils migration from bone marrow to peripheral circulation by antagonizing neutrophils migration factors (such as G-CSF) (72). Altogether, the specific mechanism of C3a/C3aR in neutrophils in the pathogenesis of NAFLD needs to further explore in the coming future.

## C3a as a Biomarker for Non-Invasive NASH Diagnosis

Although NASH is increasingly prevalent, it's hard to be diagnosed. Yet liver biopsy is recognized as the gold standard, but it is limited by its sampling bias, poor acceptability, and severe complications. Therefore, non-invasive methods are urgently needed to avoid biopsy for diagnosing NAFLD. Currently, some serum biomarkers have been widely accepted for the diagnosis of NASH such as the circulating serum levels of CK-18, the single nucleotide polymorphisms located in PNPLA3 as well as the non-coding RNAs, etc. (90). Especially, the most recent efforts concentrating on “omics” approaches (lipidomics, proteomics, and metabolomics) using high-throughput technologies have shown promising results to identify novel biomarkers of NAFLD, NASH, and advanced fibrosis (91). However, those diagnostic accuracy need to be further improved by combining other different approaches.

Complement system activation has been demonstrated in liver biopsies from patients with NAFLD compared to healthy controls. It has been confirmed that complement C3 levels increased in patients with NASH, but not in those with viral liver disease. More recently, circulating C3 levels have been demonstrated to predict the presence of NAFLD in a large cohort from general population independently of the most plausible confounders such as the presence of metabolic syndrome and obesity. In addition, some similar evidence was also confirmed in the other disease combined with NAFLD. Ursini et al. provide an important evidence for the potential role of complement C3 as a surrogate biomarker of NAFLD in a large cohort of Rheumatoid arthritis (RA) patients at the best cut-off value of 1.23 g/l for complement C3 with a sensitivity of 76% and a specificity of 64% (18). Pan et al. demonstrated the predictive role of complement C3 as a candidate biomarker for diagnosing NAFLD in chronic kidney disease (CKD) patients at the best cut-off value of 993.5 mg/L for complement C3 with a sensitivity of 63.9% and a specificity of 70.1% (17). Therefore, serum C3 may be fully used as a non-invasive diagnostic marker in the coming clinical diagnosis of NASH.

## The Potential Benefit from Antagonizing C3aR in NASH Therapy

With the increasing incidence of global NAFLD, more and more strategies are explored to prevent effectively NASH in medical treatment nowadays. It is of great significance to find the specific signal pathway leading to the occurrence of NAFLD. Intriguingly, C3aR was indicated to be a remarkable gene closely related to obesity and potential insulin resistance in the mice model intercrossed among different strains by integrated genomic analysis (92). On this base, it is speculated to be a very promising treatment through targeting C3a/C3aR in the pathogenesis of NAFLD. So far, researchers have designed a variety of small molecule antagonists for C3a/C3aR signaling pathway and verified their pharmacological effects. For example, a series of diaminoisoindoline compounds can play a significant role as C3a antagonist at the micromolar level (93). A new oral selective antagonist of C3a receptor, discovered by heterocyclic hinge control conformation, can significantly block the recruitment and activation of macrophages and neutrophils and then play the role of inhibiting the expression of inflammatory mediators (94). Also known is FLTChaAR (IC50 240 nM, Ca2+, macrophages), a significant hexapeptide C3a antagonist, which provide an important reference for the coming antagonist design (95). As C3aR antagonist obtained through high-throughput screening, Sb290157 is thought as the most promising drug for the treatment of metabolic syndrome including NAFLD in the clinical future (88, 96). In the obese rat models fed by high carbohydrate and saturated fat diet, SB 290157 can attenuate the inflammatory response by mainly controlling macrophages into adipose or liver tissue (97), which significantly reduce the obesity and body weight by effectively improving liver metabolism (27). Subsequently, it was further confirmed that the IC50s of sb290157 were 27.7 nm in RBL-C3aR cells and 28 nm human neutrophils, respectively. Most importantly, sb290157 is found to acts selectively on C3aR not C5aR or other six chemotactic G protein coupled receptors, which indicates very potential effect in the clinical application (96).

Recent evidence has elucidated C3 as a potent lipogenic hormone in the pathogenesis of NAFLD according to preclinical and translational evidence. Theoretically, initial discussions primarily relied on considerations of C3 deficiency which often leads to a broader range of susceptibilities to infections. As a matter of factor, it can be effectively avoided by developing therapies targeting C3a/C3aR, which participates in inflammatory responses such as anti COVID-19 and anti-HCC treatment (97–102). Complement C3 activation may interfere with NAFLD with at least two distinct mechanisms by enhancing adipose tissue inflammation via the local engagement of C3a and C5a receptors as well as by providing the substrate for the conversion of C3a into ASP that may exerts its effects systemically (103). Regretfully, no clinical registered trial is found to target C3a or C3aR in NASH therapy so far. Our previous studies have shown that C3a/C3aR participates in the pathogenesis of NASH even fibrosis by regulating various signaling pathways and mentioned the protective function in the C3aR^−/−^ mice model (28). Therefore, in-depth investigations and awareness of the roles of C3a/C3aR in NASH are urgently needed that will lead to a further expansion of potential indications for complement treatments in the future.

**Figure 3:**
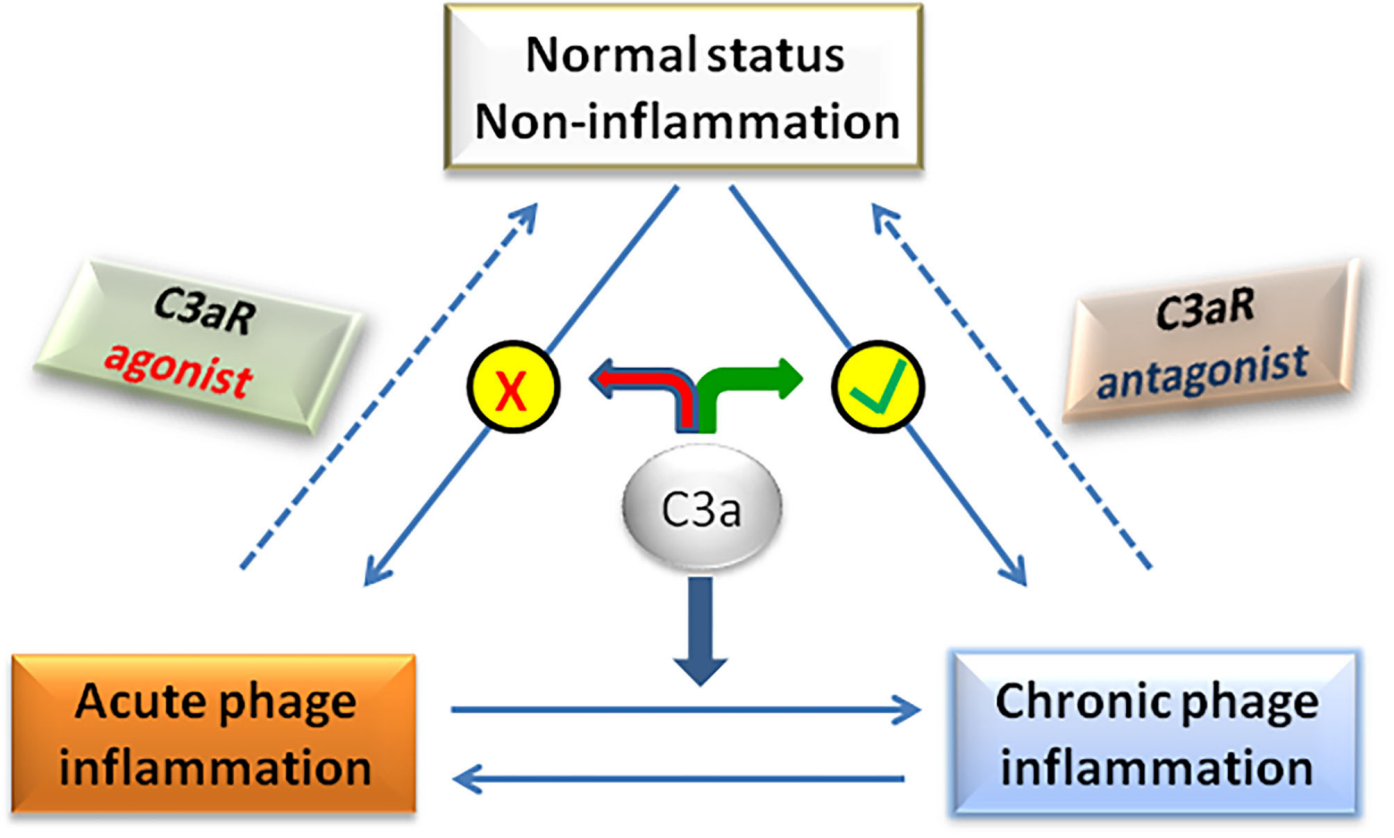
The balance of C3a actions determines the disease phenotype.

Traditionally, C3a/C3aR signaling axis plays a pro-inflammatory role in the pathogenesis of NAFLD. However, recent studies have found that in the early stage of NAFLD, C3a plays an anti-inflammatory role by preventing neutrophils from accumulating in liver tissue (58). In fact, whether C3a is pro-inflammatory or anti-inflammatory is not mutually exclusive in the pathogenesis of NAFLD, but depends on the balance between pro-inflammatory and anti-inflammatory effects of C3a, which determines the final outcome of the disease (Figure 3). For example, C3a plays a pro-inflammatory role in the NASH stage of NAFLD by activating Kupffer cells under the effect of neutrophil elastase. If neutrophils were deleted at that time, the activation of Kupffer cells was significantly delayed (104). Therefore, the dual role of C3a/C3aR signaling axis should be considered for the rational designing therapeutic strategies targeting C3a/C3aR in the effective treatment of NAFLD.

## Conclusion

Complement system is one of the most important innate immune barriers in the body, among which complement C3 is the critical component in complement cascade activation. Clinically, increased epidemiological evidence has shown that C3 is closely related to the pathogenesis of NAFLD. Serum fatty acids, adipose tissue-derived cytokines and gut derived endotoxin can take part in complement activation. After complement activation, C3 interacts with different types of liver innate immune cells, and ultimately participates in the pathogenesis of NAFLD. C3a is an important derivative from C3 when complement cascades are activated. Accumulating studies indicate that C3a plays an important role in the pathogenesis of NAFLD by interacting with its receptor C3aR. Targeted inhibition of C3aR activation is a potential strategy for the prevention and treatment of NAFLD. Although SB 290157 is an effective and selective C3aR antagonist in some experimental animal models, the preclinical and clinical evidence of SB 290157 needs to be explored to prevent dietary obesity, metabolic dysfunction and NAFLD in the coming future.

## Author Contributions

JH and XZ were involved in study design and drafted the paper. XZ supervised and reviewed the paper. All authors contributed to the article and approved the submitted version.

## Conflict of Interest

The authors declare that the research was conducted in the absence of any commercial or financial relationships that could be construed as a potential conflict of interest.

## Publisher's Note

All claims expressed in this article are solely those of the authors and do not necessarily represent those of their affiliated organizations, or those of the publisher, the editors and the reviewers. Any product that may be evaluated in this article, or claim that may be made by its manufacturer, is not guaranteed or endorsed by the publisher.

## Abbreviations

NAFLD: Non-alcoholic fatty liver disease
NASH: Non-alcoholic steatohepatitis
HCC: hepatocellular carcinoma
C3: Complement component 3
NAFL: non-alcoholic fatty liver
FDA: Food and Drug Administration
FFA: free fatty acid
DAMPs: danger associated molecular patterns
PAMPs: pathogen associated molecular patterns
MAC: membrane attack complex
MBL: mannose-binding lectin
IL: interleukin
KCs: Kupffer cells
NLR: neutrophil to lymphocyte ratio
NAS: NAFLD activity score.

## References

[1] Mantovani A. Nonalcoholic fatty liver disease (NAFLD) and risk of cardiac arrhythmias: a new aspect of the liver-heart axis. J Clin Transl Hepatol. (2017) 5:134–41. 10.14218/JCTH.2017.00005 28660151 PMC5472934

[2] Zhou F Zhou J Wang W Zhang XJ Ji YX Zhang P. Unexpected rapid increase in the burden of NAFLD in China from 2008 to 2018: a systematic review and meta-Analysis. Hepatology. (2019) 70:1119–33. 10.1002/hep.30702 31070259

[3] Younossi Z Anstee QM Marietti M Hardy T Henry L Eslam M. Global burden of NAFLD and NASH: trends, predictions, risk factors and prevention. Nat Rev Gastroenterol Hepatol. (2018) 15:11–20. 10.1038/nrgastro.2017.109 28930295

[4] Braun HA Faasse SA Vos MB. Advances in pediatric fatty liver disease: pathogenesis, diagnosis, and treatment. Gastroenterol Clin North Am. (2018) 47:949–68. 10.1016/j.gtc.2018.07.016 30337043

[5] Lung T Sakem B Risch L Würzner R Colucci G Cerny A. The complement system in liver diseases: evidence-based approach and therapeutic options. J Transl Autoimmun. (2019) 2:100017. 10.1016/j.jtauto.2019.100017 32743505 PMC7388403

[6] Sharma M Mitnala S Vishnubhotla RK Mukherjee R Reddy DN Rao PN. The riddle of nonalcoholic fatty liver disease: progression from nonalcoholic fatty liver to nonalcoholic steatohepatitis. J Clin Exp Hepatol. (2015) 5:147–58. 10.1016/j.jceh.2015.02.002 26155043 PMC4491606

[7] Ricklin D Hajishengallis G Yang K Lambris JD. Complement: a key system for immune surveillance and homeostasis. Nat Immunol. (2010) 11:785–97. 10.1038/ni.1923 20720586 PMC2924908

[8] Reis ES Mastellos DC Hajishengallis G Lambris JD. New insights into the immune functions of complement. Nat Rev Immunol. (2019) 19:503–16. 10.1038/s41577-019-0168-x 31048789 PMC6667284

[9] Thorgersen EB Barratt-Due A Haugaa H Harboe M Pischke SE Nilsson PH. The role of complement in liver injury, regeneration, and transplantation. Hepatology. (2019) 70:725–36. 10.1002/hep.30508 30653682 PMC6771474

[10] Wagner E Frank MM. Therapeutic potential of complement modulation. Nat Rev Drug Discov. (2010) 9:43–56. 10.1038/nrd3011 19960015

[11] Ju C Tacke F. Hepatic macrophages in homeostasis and liver diseases: from pathogenesis to novel therapeutic strategies. Cell Mol Immunol. (2016) 13:316–27. 10.1038/cmi.2015.104 26908374 PMC4856798

[12] Yesilova Z Ozata M Oktenli C Bagci S Ozcan A Sanisoglu SY. Increased acylation stimulating protein concentrations in nonalcoholic fatty liver disease are associated with insulin resistance. Am J Gastroenterol. (2005) 100:842–9. 10.1111/j.1572-0241.2005.40838.x 15784030

[13] Wlazlo N van Greevenbroek MM Ferreira I Jansen EH Feskens EJ van der Kallen CJ. Activated complement factor 3 is associated with liver fat and liver enzymes: the CODAM study. Eur J Clin Invest. (2013) 43:679–88. 10.1111/eci.12093 23586841

[14] Jia Q Li C Xia Y Zhang Q Wu H Du H. Association between complement C3 and prevalence of fatty liver disease in an adult population: a cross-sectional study from the tianjin chronic low-grade systemic inflammation and health (TCLSIHealth) cohort study. PLoS ONE. (2015) 10:e0122026. 10.1371/journal.pone.0122026 25856141 PMC4391843

[15] Xu C Chen Y Xu L Miao M Li Y Yu C. Serum complement C3 levels are associated with nonalcoholic fatty liver disease independently of metabolic features in Chinese population. Sci Rep. (2016) 6:23279. 10.1038/srep23279 27029598 PMC4814815

[16] Rensen SS Slaats Y Driessen A Peutz-Kootstra CJ Nijhuis J Steffensen R. Activation of the complement system in human nonalcoholic fatty liver disease. Hepatology. (2009) 50:1809–1817. 10.1002/hep.23228 19821522

[17] Pan B Wan X Ma M Cao C. Complement C3 and nonalcoholic fatty liver disease in chronic kidney disease patients: a pilot study. Kidney Blood Press Res. (2020) 45:61–9. 10.1159/000504172 31968339

[18] Ursini F Russo E Mauro D Abenavoli L Ammerata G Serrao A. Complement C3 and fatty liver disease in rheumatoid arthritis patients: a cross-sectional study. Eur J Clin Invest. (2017) 47:728–35. 10.1111/eci.12798 28796299

[19] Himoto T Hirakawa E Fujita K Sakamoto T Nomura T Morishita A. Complement component 3 as a surrogate hallmark for metabolic abnormalities in patients with chronic hepatitis C. Ann Clin Lab Sci. (2019) 49:79–88. 30814081

[20] Vaisar T Pennathur S Green PS Gharib SA Hoofnagle AN Cheung MC. Shotgun proteomics implicates protease inhibition and complement activation in the antiinflammatory properties of HDL. J Clin Invest. (2007) 117:746–56. 10.1172/JCI26206 17332893 PMC1804352

[21] Choy LN Rosen BS Spiegelman BM. Adipsin and an endogenous pathway of complement from adipose cells. J Biol Chem. (1992) 267:12736–41 10.1016/S0021-9258(18)42338-1 1618777

[22] Persson L Borén J Robertson AK Wallenius V Hansson GK Pekna M. Lack of complement factor C3, but not factor B, increases hyperlipidemia and atherosclerosis in apolipoprotein E–/– low-density lipoprotein receptor–/– mice. Arterioscler Thromb Vasc Biol. (2004) 24:1062–7. 10.1161/01.ATV.0000127302.24266.40 15059809

[23] Brunt EM. Pathology of nonalcoholic fatty liver disease. Nat Rev Gastroenterol Hepatol. (2010) 7:195–203. 10.1038/nrgastro.2010.21 20195271

[24] Meli R. Raso M Calignano G. Role of innate immune response in non-alcoholic fatty liver disease: metabolic complications and therapeutic tools. Front Immunol. (2014) 5:177. 10.3389/fimmu.2014.00177 24795720 PMC4005965

[25] Malhi H Gores GJ. Molecular mechanisms of lipotoxicity in nonalcoholic fatty liver disease. Semin Liver Dis. (2008) 28:360–9. 10.1055/s-0028-1091980 18956292 PMC2908270

[26] Cianflone K Xia Z Chen LY. Critical review of acylation-stimulating protein physiology in humans and rodents. Biochem Biophys Acta. (2003) 1609:127–43. 10.1016/S0005-2736(02)00686-7 12543373

[27] Lim J Iyer A Suen JY Seow V Reid RC Brown L. C5aR and C3aR antagonists each inhibit diet-induced obesity, metabolic dysfunction, and adipocyte and macrophage signaling. FASEB J. (2013) 27:822–31. 10.1096/fj.12-220582 23118029

[28] Han J Zhang X Lau JK Fu K Lau HC Xu W. Bone marrow-derived macrophage contributes to fibrosing steatohepatitis through activating hepatic stellate cells. J Pathol. (2019) 248:488–500. 10.1002/path.5275 30945293 PMC6767065

[29] Ricklin D Reis ES Mastellos DC Gros P Lambris JD. Complement component C3 - the “Swiss Army Knife” of innate immunity and host defense. Immunol Rev. (2016) 274:33–58 10.1111/imr.12500 27782325 PMC5427221

[30] Markiewski MM Lambris JD. The role of complement in inflammatory diseases from behind the scenes into the spotlight. Am J Pathol. (2007) 171:715–27. 10.2353/ajpath.2007.070166 17640961 PMC1959484

[31] Gerard N P Gerard C. Complement in allergy and asthma. Curr Opin Immunol. (2002) 14:705–8. 10.1016/S0952-7915(02)00410-7 12413519

[32] Mizutani N Nabe T Yoshino S. Complement C3a regulates late asthmatic response and airway hyperresponsiveness in mice. J Immunol. (2009) 183:4039–46. 10.4049/jimmunol.0901468 19684087

[33] Hutamekalin P Takeda K Tani M Tsuga Y Ogawa N Mizutani N. Effect of the C3a-receptor antagonist SB 290157 on anti-OVA polyclonal antibody-induced arthritis. J Pharmacol Sci. (2010) 112:56–63. 10.1254/jphs.09180FP 20051658

[34] Kildsgaard J Hollmann TJ Matthews KW Bian K Murad F Wetsel RA. Cutting edge: targeted disruption of the C3a receptor gene demonstrates a novel protective anti-inflammatory role for C3a in endotoxinshock. J Immunol. (2000) 165:5406–9. 10.4049/jimmunol.165.10.5406 11067891

[35] Jacob A Bao L Brorson J Quigg RJ Alexander JJ. C3aR inhibition reduces neurodegeneration in experimental lupus. Lupus. (2010) 19:73–82. 10.1177/0961203309348978 19900981 PMC2952506

[36] Mamane Y Chung Chan C Lavallee G Morin N Xu LJ Huang J. The C3a anaphylatoxin receptor is a key mediator of insulin resistance and functions by modulating adipose tissue macrophage infiltration and activation. Diabetes. (2009) 58:2006–17. 10.2337/db09-0323 19581423 PMC2731537

[37] Proctor LM Arumugam TV Shiels I Reid RC Fairlie DP Taylor SM. Comparative anti-inflammatory activities of antagonists to C3a C5a receptors in a rat model of intestinal ischaemia/reperfusion injury. Br J Pharmacol. (2004) 142:756–64. 10.1038/sj.bjp.0705819 15159277 PMC1575041

[38] Chazin WJ Hugli TE Wright PE. 1H NMR studies of human C3a anaphylatoxin in solution: sequential resonance assignments, secondary structure, and global fold. Biochemistry. (1988) 27:9139–48. 10.1021/bi00426a011 3266557

[39] Hugli TE Erickson BW. Synthetic peptides with the biological activities and specificity of human C3a anaphylatoxin. Proc Natl Acad Sci USA. (1977) 74:1826–30. 10.1073/pnas.74.5.1826 266705 PMC431017

[40] Chang JY Lin CC Salamanca S Pangburn MK Wetsel RA. Denaturation and unfolding of human anaphylatoxin C3a: an unusually low covalent stability of its native disulfide bonds. Arch Biochem Biophys. (2008) 480:104–10. 10.1016/j.abb.2008.09.013 18854167 PMC2636726

[41] Langkabel P Zwirner J Oppermann M. Ligand-induced phosphorylation of anaphylatoxin receptors C3aR and C5aR is mediated by G proteincoupled receptor kinases. Eur J Immunol. (1999) 29:3035–46. 10.1002/(SICI)1521-4141(199909)29:09<3035::AID-IMMU3035>3.0.CO;2-Z 10508278

[42] Monsinjon T Gasque P Chan P Ischenko A Brady JJ Fontaine MC. Regulation by complement C3a and C5a anaphylatoxins of cytokine production in human umbilical vein endothelial cells. FASEB J. (2003) 17:1003–14. 10.1096/fj.02-0737com 12773483

[43] Sayah S Jauneau C Patte C Tonon MC Vaudry H Fontaine M. Two different transduction pathways are activated by C3a and C5a anaphylatoxins on astrocytes. Brain Res Mol Brain Res. (2003) 112:53–60. 10.1016/S0169-328X(03)00046-9 12670702

[44] Shinjyo N Stahlberg A Dragunow M Pekny M Pekna M. Complement-derived anaphylatoxin C3a regulates in vitro differentiation and migration of neural progenitor cells. Stem Cells. (2009) 27:2824–32. 10.1002/stem.225 19785034

[45] Ruan B Li X Winkler A Cunningham KM Kuai J Greco RM. Complement C3a,CpG oligos, and DNA/C3a complex stimulate IFN-a production in a receptor for advanced glycation end product-dependent manner. J Immunol. (2010) 185:4213–22. 10.4049/jimmunol.1000863 20817881

[46] Hannedouche S Beck V Leighton-Davies J Beibel M Roma G Oakeley EJ. Identification of the C3a receptor (C3aR1) as the target of the VGF-derived peptide TLQP-21 in rodent cells. J Biol Chem. (2013) 288:27434–43. 10.1074/jbc.M113.497214 23940034 PMC3779738

[47] Cero C Vostrikov VV Verardi R Severini C Gopinath T Braun PD. The TLQP-21 peptide activates the G-protein-coupled receptor C3aR1 via a folding-upon-binding mechanism. Structure. (2014) 22:1744–53. 10.1016/j.str.2014.10.001 25456411 PMC4353613

[48] Xie J Me'ndez J Me'ndez-Valenzuela V Aguilar-Hernández MM. Cellular signaling of the receptor for advanced glycation end products (RAGE). Cell Signal. (2013) 25:2185–97. 10.1016/j.cellsig.2013.06.013 23838007

[49] Kazankov K Jørgensen SMD Thomsen KL Møller HJ Vilstrup H George J. The role of macrophages in nonalcoholic fatty liver disease and nonalcoholic steatohepatitis. Nat Rev Gastroenterol Hepatol. (2019) 16:145–59. 10.1038/s41575-018-0082-x 30482910

[50] Devisscher L Verhelst X Colle I Van Vlierberghe H Geerts A. The role of macrophages in obesity-driven chronic liver disease. J Leukocyte Biolog. (2016) 99:693–8. 10.1189/jlb.5RU0116-016R 26936934

[51] Baffy G. Kupffer cells in non-alcoholic fatty liver diseases: the emerging view. J Hepatol. (2009) 51:212–23. 10.1016/j.jhep.2009.03.008 19447517 PMC2694233

[52] Marra F Tacke F. Roles for chemokines in liver disease. Gastroenterology. (2014) 147:577–94. 10.1053/j.gastro.2014.06.043 25066692

[53] Miura K Yang L van Rooijen N Ohnishi H Seki E. Hepatic recruitment of macrophages promotes nonalcoholic steatohepatitis through CCR2. Am J Physiol Gastrointest Liver Physiol. (2012) 302:G1310–321. 10.1152/ajpgi.00365.2011 22442158 PMC3378163

[54] Tosello-Trampont AC1 Landes SG Nguyen V Novobrantseva TI Hahn YS. Kuppfer cells trigger nonalcoholic steatohepatitis development in diet-induced mouse model through tumor necrosis factor-a production. J Bio Chem. (2012) 287:40161–72. 10.1074/jbc.M112.417014 23066023 PMC3504730

[55] Duffield JS Forbes SJ Constandinou CM Clay S Partolina M Vuthoori S. Selective depletion of macrophages reveals distinct, opposing roles during liver injury and repair. J Clin Invest. (2005) 115:56–65. 10.1172/JCI200522675 15630444 PMC539199

[56] Sunami Y Leithäuser F Gul S Fiedler K Güldiken N Espenlaub S. Hepatic activation of IKK/NFκB signaling induces liver fibrosis via macrophage-mediated chronic inflammation. Hepatology. (2012) 56:1117–28. 10.1002/hep.25711 22407857

[57] Banda NK Hyatt S Antonioli AH White JT Glogowska M Takahashi K. Role of C3a receptors, C5a receptors, and complement protein C6 deficiency in collagen antibody-induced arthritis in mice. J Immunol. (2012) 188:1469–78. 10.4049/jimmunol.1102310 22205026 PMC3262949

[58] Coulthard LG Woodruff TM. Is the complement activation product C3a a proinflammatory molecule? Re-evaluating the evidence and the myth. J Immunol. (2015) 194:3542–48. 10.4049/jimmunol.1403068 25848071

[59] Takabayashi T Vannier E Clark BD Margolis NH Dinarello CA Burke JF. A new biologic role for C3a and C3a desArg: regulation of TNF-alpha and IL-1 beta synthesis. J Immunol. (1996) 156:3455–60. 8617973

[60] Takabayashi T Vannier E Burke JF Tompkins RG Gelfand JA Clark BD. Both C3a and C3a(desArg) regulate interleukin-6 synthesis in human peripheral blood mononuclear cells. J Infect Dis. (1998) 177:1622–8. 10.1086/515316 9607842

[61] Monsinjon T Gasque P Ischenko A Fontaine M. C3a binds to the seven transmembrane anaphylatoxin receptor expressed by epithelial cells and triggers the production of IL-8. FEBS Lett. (2001) 487:339–46. 10.1016/S0014-5793(00)02320-6 11163355

[62] Fischer WH Jagels MA Hugli TE. Regulation of IL-6 synthesis in human peripheral blood mononuclear cells by C3a and C3a (desArg). J. Immunol. (1999) 162:453–9. 9886419

[63] Asgari E Le Friec G Yamamoto H Perucha E Sacks SS Köhl J. C3a modulates IL-1b secretion in human monocytes by regulating ATP efflux and subsequent NLRP3 inflammasome activation. Blood. (2013) 122:3473–81. 10.1182/blood-2013-05-502229 23878142

[64] Malik A Thanekar U Amarachintha S Mourya R Nalluri S Bondoc A. “Complimenting the complement”: mechanistic insights and opportunities for therapeutics in hepatocellular carcinoma. Front Oncol. (2021) 10:627701–31. 10.3389/fonc.2020.627701 33718121 PMC7943925

[65] Xu Y Huang Y Xu W Zheng X Yi X Huang L. Activated hepatic stellate cells (HSCs) exert immunosuppressive effects in hepatocellular carcinoma by producing complement C3. Onco Targets Ther. (2020) 13:1497–505. 10.2147/OTT.S234920 32110047 PMC7035898

[66] Weiskirchen R Tacke F. Cellular and molecular functions of hepatic stellate cells in inflammatory responses and liver immunology. Hepatobiliary Surg Nutr. (2014) 3:344–63. 10.3978/j.issn.2304-3881.2014.11.03 25568859 PMC4273119

[67] Maher JJ Leon P Ryan JC. Beyond insulin resistance: innate immunity in nonalcoholic steatohepatitis. Hepatology. (2008) 48:670–8. 10.1002/hep.22399 18666225 PMC3592568

[68] Hammerich L Heymann F Tacke F. Role of IL-17 and Th17 cells in liver diseases. Clin Dev Immunol. (2011) 2011:345803. 10.1155/2011/345803 21197451 PMC3010664

[69] Tang Y Bian Z Zhao L Liu Y Liang S Wang Q. Interleukin-17 exacerbates hepatic steatosis and inflammation in non-alcoholic fatty liver disease. Clin Exp Immunol. (2011) 166:281–90. 10.1111/j.1365-2249.2011.04471.x 21985374 PMC3219903

[70] Rau M Schilling AK Meertens J Hering I Weiss J Jurowich C. Progression from nonalcoholic fatty liver to nonalcoholic steatohepatitis is marked by a higher frequency of Th17 cells in the liver and an increased Th17/resting regulatory T cell ratio in peripheral blood and in the liver. J Immunol. (2016) 196:97–105. 10.4049/jimmunol.1501175 26621860

[71] Li J Qiu SJ She WM Wang FP Gao H Li L. Significance of the balance between regulatory T (Treg) and T helper 17 (Th17) cells during hepatitis B virus related liver fibrosis. PLoS ONE. (2012) 7:e39307. 10.1371/journal.pone.0039307 22745730 PMC3380028

[72] Liu Y She W Wang F Li J Wang J Jiang W. 3, 3'-diindolylmethane alleviates steatosis and the progression of NASH partly through shifting the imbalance of Treg/Th17 cells to Treg dominance. Int Immunopharmacol. (2014) 23:489–98. 10.1016/j.intimp.2014.09.024 25281898

[73] Lim H Kim Y Drouin S Mueller-Ortiz S Yun K Morschl E. Negative regulation of pulmonary Th17 responses by C3a anaphylatoxin during allergic inflammation in mice. PLoS ONE. (2012) 7:e52666. 10.1371/journal.pone.0052666 23285141 PMC3527591

[74] Drouin S Corry M Hollman T Brodeur S Bryce P Lu B. Absence of the complement anaphylatoxin C3a receptor suppresses Th2 effector functions in a murine model of pulmonary allergy. J Immunol. (2002) 169:5926–33. 10.4049/jimmunol.169.10.5926 12421977

[75] Martin U Bock D Arseniev L Tornetta MA Ames RS Bautsch W. The human C3a receptor is expressed on neutrophils and monocytes, but not on B or T lymphocytes. J Exp Med. (1997) 186:199–207. 10.1084/jem.186.2.199 9221749 PMC2198980

[76] Strainic M Shevach E An F Lin F Medof ME. Absence of signaling into CD4+ cells via C3aR and C5aR enables autoinductive TGF-b1 signaling and induction of Foxp3+ regulatory T cells. Nat Immunol. (2013) 14:162–171. 10.1038/ni.2499 23263555 PMC4144047

[77] Liszewski MK Kolev M Le Friec G Leung M Bertram PG Fara AF. Intracellular complement activation sustains T cell homeostasis and mediates effector differentiation. Immunity. (2013) 39:1143–57. 10.1016/j.immuni.2013.10.018 24315997 PMC3865363

[78] Kwan W van der Touw W Paz-Artal E Li MO Heeger PS. Signaling through C5a receptor and C3a receptor diminishes function of murine natural regulatory T cells. J. Exp. Med. (2013) 210:257–68. 10.1084/jem.20121525 23382542 PMC3570105

[79] Strainic M Liu J Huang D An F Lalli PN Muqim N. Locally produced complement fragments C5a and C3a provide both costimulatory and survival signals to naive CD4+ T cells. Immunity. (2008) 28:425–35. 10.1016/j.immuni.2008.02.001 18328742 PMC2646383

[80] Kawamoto S Yalcindag A Laouini D Brodeur S Bryce P Lu B. The anaphylatoxin C3a downregulates the Th2 response to epicutaneously introduced antigen. J Clin Invest. (2004) 114:399–407. 10.1172/JCI200419082 15286806 PMC484971

[81] Nati M Haddad D Birkenfeld AL Koch CA Chavakis T Chatzigeorgiou A. The role of immune cells in metabolism-related liver inflammation and development of non-alcoholic steatohepatitis (NASH). Rev Endocr Metab Disord. (2016) 17:29–39. 10.1007/s11154-016-9339-2 26847547

[82] Cai JJ Zhang XJ Li HL. The role of innate immune cells in nonalcoholic steatohepatitis. Hepatology. (2019) 70:1026–37. 10.1002/hep.30506 30653691

[83] Ou R Liu J Lv M Wang J Wang J Zhu L. Neutrophil depletion improves diet-induced non-alcoholic fatty liver disease in mice. Endocrine. (2017) 57:72–82. 10.1007/s12020-017-1323-4 28508193

[84] Yilmaz H Yalcin KS Namuslu M Celik HT Sozen M Inan O. Neutrophil-lymphocyte ratio (NLR) could be better predictor than C-reactive protein (CRP) for liver fibrosis in non-alcoholic steatohepatitis(NASH). Ann Clin Lab Sci. (2015) 45:278–86. 26116591

[85] Khoury T Mari A Nseir W Kadah A Sbeit W Mahamid M. Neutrophil-to-lymphocyte ratio is independently associated with inflammatory activity and fibrosis grade in nonalcoholic fatty liver disease. Eur J Gastroenterol Hepatol. (2019) 31:1110–5. 10.1097/MEG.0000000000001393 30888972

[86] Hatting M Tacke F. From NAFLD to HCC: is IL-17 the crucial link? Hepatology. (2017) 65:739–41. 10.1002/hep.28934 28012256

[87] Alkhouri N Morris-Stiff G Campbell C Lopez R Tamimi TA Yerian L. Neutrophil to lymphocyte ratio: a new marker for predicting steatohepatitis and fibrosis in patients with nonalcoholic fatty liver disease. Liver Int. (2012) 32:297–302. 10.1111/j.1478-3231.2011.02639.x 22097893

[88] Bettelli E Carrier Y Gao W Korn T Strom TB Oukka M. Reciprocal developmental pathways for the generation of pathogenic effector TH17 and regulatory T cells. Nature. (2006) 441:235–8. 10.1038/nature04753 16648838

[89] Geginat J Paroni M Maglie S Alfen JS Kastirr I Gruarin P. Plasticity of human CD4 T cell subsets. Front Immunol. (2014) 5:630. 10.3389/fimmu.2014.00630 25566245 PMC4267263

[90] Castera L Friedrich-Rust M Loomba R. Noninvasive assessment of liver disease in patients with nonalcoholic fatty liver disease. Gastroenterology. (2019) 156:1264–81.e4. 10.1053/j.gastro.2018.12.036 30660725 PMC7505052

[91] Pirola CJ Sookoian S. Multiomics biomarkers for the prediction of nonalcoholic fatty liver disease severity. World J Gastroenterol. (2018) 24:1601–15. 10.3748/wjg.v24.i15.1601 29686467 PMC5910543

[92] Schadt EE Lamb J Yang X Zhu J Edwards S Guhathakurta D. An integrative genomics approach to infer causal associations between gene expression and disease. Nat Genet. (2005) 37:710–7. 10.1038/ng1589 15965475 PMC2841396

[93] Grant EB Guiadeen D Singer M Argentieri D Hlasta DJ Wachter M. Design, synthesis, and biological activity of diiminoisoindolines as complement component 3a antagonists. Bioorg Med Chem Lett. (2001) 11:2817–20. 10.1016/S0960-894X(01)00522-4 11597407

[94] Lohman RJ Hamidon JK Reid R C Rowley JA Yau MK Halili MA. Exploiting a novel conformational switch to control innate immunity mediated by complement protein C3a. Nat Commun. (2017) 8:351–66. 10.1038/s41467-017-00414-w 28839129 PMC5570900

[95] Scully CC Blakeney JS Singh R Hoang HN Abbenante G Reid RC. Selective hexapeptide agonists and antagonists for human complement C3a receptor. J Med Chem. (2010) 53:4938–48. 10.1021/jm1003705 20527893

[96] Ames RS Lee D Foley JJ Jurewicz AJ Tornetta MA Bautsch W. Identification of a selective nonpeptide antagonist of the anaphylatoxin C3a receptor that demonstrates anti-inflammatory activity in animal models. J Immunol. (2001) 166:6341–8. 10.4049/jimmunol.166.10.6341 11342658

[97] Ajona D Ortiz-Espinosa S Pio R. Complement anaphylatoxins C3a and C5a: emerging roles in cancer progression and treatment. Semin Cell Dev Biol. (2019) 85:153–63. 10.1016/j.semcdb.2017.11.023 29155219

[98] Pio R Ajona D Lambris JD. Complement inhibition in cancer therapy. Semin Immunol. (2013) 25:54–64. 10.1016/j.smim.2013.04.001 23706991 PMC3733085

[99] Sinkovits G Mezo B Réti M Müller V Iványi Z Gál J. Complement overactivation and consumption predicts in-hospital mortality in SARS-CoV-2 infection. Front Immunol. (2021) 12:663187–210. 10.3389/fimmu.2021.663187 33841446 PMC8027327

[100] Mastellos DC Pires da Silva BGP Fonseca BAL Fonseca NP Auxiliadora-Martins M Mastaglio S. Complement C3 vs C5 inhibition in severe COVID-19: early clinical findings reveal differential biological efficacy. Clin Immunol. (2020) 220:108598–608. 10.1016/j.clim.2020.108598 32961333 PMC7501834

[101] Ursini F Abenavoli L. The emerging role of complement C3 as a biomarker of insulin resistance and cardiometabolic diseases: preclinical and clinical evidence. Rev Recent Clin Trials. (2018) 13:61–8. 10.2174/1574887112666171128134552 29189176

[102] Mastaglio S Ruggeri A Risitano AM Angelillo P Yancopoulou D Mastellos DC. The first case of COVID-19 treated with the complement C3 inhibitor AMY-101. Clin Immunol. (2020) 215:108450–4. 10.1016/j.clim.2020.108450 32360516 PMC7189192

[103] Yuan M Konstantopoulos N Lee J Hansen L Li ZW Karin M. Reversal of obesity- and diet-induced insulin resistance with salicylates or targeted disruption of Ikkbeta. Science. (2001) 293:1673–7. 10.1126/science.1061620 11533494

[104] Zang S Wang L Ma X Zhu G Zhuang Z Xun Y. Neutrophils play a crucial role in the early stage of nonalcoholic steatohepatitis via neutrophil elastase in mice. Cell Biochem Biophys. (2015) 73:479–87. 10.1007/s12013-015-0682-9 27352342

